# Seborrheic Dermatitis: Exploring the Complex Interplay with *Malassezia*

**DOI:** 10.3390/ijms26062650

**Published:** 2025-03-14

**Authors:** Francesca Piacentini, Emanuela Camera, Anna Di Nardo, Maria Lucia Dell’Anna

**Affiliations:** Laboratory of Cutaneous Physiopathology and Integrated Center of Metabolomics Research, San Gallicano Dermatological Institute IRCCS, 00144 Rome, Italy; francesca.piacentini23@ifo.it (F.P.); emanuela.camera@ifo.it (E.C.)

**Keywords:** seborrheic dermatitis, *Malassezia*, cytokine, inflammation

## Abstract

Seborrheic dermatitis (SD) is a chronic inflammatory skin condition often involving the sebaceous-rich areas, characterized by erythematous scaly lesions. It is frequently observed in individuals with immune dysregulation, suggesting the interplay between the immune system and disease development. An altered immune environment leads to an exaggerated inflammatory response with the activation of innate immunity, involving the participation of mast cells, γδ T cells, and the NOD–LRR–pyrin-domain-containing protein 3 (NLRP3) inflammasome. This review aims to assess the complex relationship between *Malassezia* and the immune system in the pathogenesis of SD. We will explore how an impaired immune response predisposes the skin to *Malassezia* overgrowth and infection. We will examine the role of adaptive immunity, particularly T helper cells, in driving chronic inflammation in SD. All actors involved, whether part of innate or adaptive immunity, are responsible for the release of pro-inflammatory cytokines, which contribute to the progression of the disease. Therapeutic strategies aimed at the modulation of the immune response in SD have been tested in clinical trials evaluating the efficacy of immunomodulatory treatments in the management of SD. This review synthesizes insights from immunological studies and clinical trials to present an in-depth analysis of the immune mechanisms underpinning SD, thereby proposing targeted therapeutic strategies for its management.

## 1. Introduction

Seborrheic dermatitis (SD) is a chronic inflammatory skin disease that predominantly affects the seborrheic areas of the body, including the scalp, face (nasolabial folds, ears, and eyebrows), and the upper part of the trunk [1]. The condition manifests more frequently in men. It shows a higher prevalence during specific life stages: infancy within the first three months of life, adolescence and young adulthood, and after the age of 50 years. SD affects approximately 1% to 3% of immunocompetent adults [2,3,4]. SD is more common in patients with neurologic and psychiatric conditions, including Parkinson’s disease [5,6]. The etiology of SD remains a topic of considerable debate in the dermatological field. The current understanding of the pathogenesis of SD points to several factors. Genetic predisposition plays a significant role, making some individuals more susceptible to disease development. Environmental and behavioral factors, such as stress, climatic variations (particularly humidity and cold), sebum excess, and poor hygiene habits, may influence the onset and severity of SD. Furthermore, alterations in the skin barrier are frequently observed in individuals with SD. Recent studies have focused on the alteration of the skin microbiome and have found that changes in microbial communities are associated with the pathogenesis of SD.

Seborrheic Dermatitis: Malassezia, Immunity, or Both?

The leading theory on SD etiology postulates a potential association between the fungal genus *Malassezia* and SD [7].

*Malassezia* is a lipophilic organism that colonizes lipid-rich areas, such as the skin, and metabolizes sebum components [4]. Recent studies have shown that *Malassezia* are common commensals on the skin of 75% to 98% of healthy adults. Over millions of years, *Malassezia* has co-evolved with mammals, acquiring specialized adaptations such as a reduction in fatty acid synthase (FAS) genes and an increase in lipid hydrolases, allowing it to thrive in various human body niches. Dysbiosis, caused by factors like antibiotics, immunosuppression, and environmental changes, can lead to its colonization [8].

Hamido et al. conducted a significant study on the epidemiological prevalence of *Malassezia* in various skin diseases. They found that *M. furfur* was most prevalent in pityriasis versicolor and SD lesions, particularly on the scalp, while *M. sympodialis* was dominant in atopic dermatitis lesions. In healthy individuals, *M. furfur*, *M. globosa*, and *M. sympodialis* were present at lower frequencies [9].

Genetic diversity of *Malassezia* spp. has been found in SD patients. *M. restricta* and *M. arunalokei* exhibited less genetic variation, while *M. globosa* showed higher genetic diversity [10]. A recent study of Hiruma and coworkers identified a novel species (*M. polysorbatinonusus*) occurring in a Japanese patient with SD. They molecularly characterized the species, suggesting its similarity to another *Malassezia* spp. (e.g., *M. yamatoensis*) already associated with the disease, even if there were some different growth features [11,12].

Recent studies have highlighted significant skin dysbiosis in SD patients, with an increased relative abundance of *M. restricta* and *Staphylococcus* spp., which may contribute to disease by disrupting the skin barrier and exacerbating inflammation. Furthermore, SD-affected skin showed reduced fungal diversity, increased *M. restricta* abundance, and a higher *M. restricta*/*C. acnes* ratio compared to healthy skin, alongside increased *Staphylococcus* spp. and reduced *C. acnes* abundances [13,14].

Chang et al. provide a critical re-evaluation of the role of *Malassezia* in SD. While previous studies have suggested a central role for *Malassezia* in SD, emerging evidence now suggests that immune dysregulation and skin barrier dysfunction are central to the pathogenesis of the disease, with *Malassezia* acting more as a secondary factor [15]. This shift in perspective is significant for our review, as it emphasizes the need to consider the complex interplay between *Malassezia* and the host immune system. These findings align with our review’s goal of emphasizing the immune system’s role in SD pathogenesis, alongside microbial factors.

Understanding the relationship between *Malassezia* and the patient’s inadequate immune response is essential in the pathogenesis of SD. Although *Malassezia* is a ubiquitous commensal organism on the skin, the distinguishing element in patients with SD is the exaggerated or aberrant immune reactivity towards this yeast. The immune dysregulation serves as both the initiating and perpetuating factor for the chronic inflammation and clinical manifestations [16]. SD is more prevalent in immunocompromised patients, highlighting the critical role of immune mechanisms in the disease’s development. This is particularly evident in individuals with Human Immunodeficiency Virus/Acquired Immunodeficiency Syndrome (HIV/AIDS), where SD tends to be more severe and more resistant to treatment, worsening as the disease progresses [17]. The immune system of individuals with HIV is ineffective in eradicating the yeast due to immune dysregulation, causing excessive yeast proliferation and marked inflammation [18]. The hypo-responsiveness leads to the worsening of SD symptoms, suggesting that *Malassezia* itself is not the direct cause of SD, but rather it is linked to the type and extent of the immune response to it. This is further supported by the observation that antifungal therapies improve SD clinically, with no definitive resolution [1]. Rather than through a biunivocal mechanism, *Malassezia* plays a pathogenic role when the immune response is deranged. Both the onset and persistence of the disease are the result of dynamic processes involving the immune and the inflammatory responses. This review aims to examine the role of the immune system in SD, focusing on how alterations in the immune response can facilitate the colonization and proliferation of *Malassezia*.

## 2. Skin Barrier

The skin is the body’s primary defense against external threats and is organized as a multilayered tissue formed by the epidermis, the dermis, and the hypodermis. The epidermis consists of keratinocytes in the basal, spinosum, granulosum, and corneum layers, each one characterized by a specific cell renewal and differentiation program aimed at the formation of a competent barrier function. Specialized cells such as melanocytes and Langerhans cells are also present in the epidermis. The impermeable barrier in the epidermis plays a crucial role in the pathophysiology of several skin disorders. Conditions characterized by significant barrier disruption and inflammation include irritant and allergic contact dermatitis, atopic dermatitis, and psoriasis [19].

Presumably, the impairment of the skin barrier is involved in the pathogenesis of SD [20], given the potential for external triggers like *Malassezia* spp. and its metabolites to penetrate the skin [21].

Park et al. examined the growth of *M. furfur* under varying pH conditions and found optimal growth at pH 6. Lipid content was highest at pH 6 and lowest at pH 7, suggesting that pH influences lipid metabolism and extracellular lipase activity. Additionally, lipids produced under acidic conditions may play a role in modulating the inflammatory response in skin cells [22].

The lipid composition and the organization of the epidermal matrix are crucial in a skin barrier opposed to the access of microbes [23]. The in-depth characterization of cutaneous barrier function can be achieved through transepidermal water loss (TEWL) measurements combined with ceramide profiling via lipidomic analysis. Such detailed assessments may provide new insights into the involvement of endogenous determinants of SD. Accordingly, the barrier–inflammation axis may play a significant role in mild-to-moderate SD, indicating that it may be more critical than changes in the microbiome [24]. The results corroborate and extend current knowledge regarding the roles of inflammation and microbial factors in SD while enhancing our understanding of barrier dysfunction. Although improving skin barrier function has been suggested as a potential adjunctive therapy for SD [24], current treatment strategies primarily focus on anti-inflammatory and antifungal approaches [25].

Considering these findings, it seems reasonable to posit that disruptions in the skin barrier and immunological mechanisms reinforce each other, initiating and perpetuating skin lesions.

## 3. Sebaceous Glands

The sebaceous glands (SGs) are holocrine glands that play a crucial role in the pilosebaceous unit and are important for skin homeostasis. They are key in areas such as cell biology, lipid signaling, and hormone regulation. Disruptions in the SGs’ function, resulting into atrophy and modified lipid profiles, can impair the skin’s barrier [26].

SGs have been demonstrated to play an important role in the etiology of numerous dermatological conditions [27,28] and likely contribute to the development of SD [29]. The lipid film on the skin’s surface consists of lipids derived from the disintegration of sebocytes that blend with the lipid matrix in the stratum corneum [30].

Sebum lipids are composed by weight of approximately 50–60% triglycerides and fatty acids, 20–25% wax esters, 12–20% squalene, and 2% cholesterol [31]. Upon secretion of sebum onto the skin’s surface, bacterial and yeast lipases hydrolyze triglycerides into free fatty acids (FFAs) [32].

Lipase activity has been identified in multiple *Malassezia* species. The isolated lipases from *M. globosa* cells present specificity for diolein, while intact cells show activity against both diolein and triolein, suggesting the presence of multiple lipases. A specific lipase has been purified, and its gene (LIP1) identified. Expression of this gene has been confirmed in samples from the human scalp, providing a starting point in understanding the role of lipid metabolism in SD [33].

Based on the *Malassezia*’s capacity to metabolize human triglycerides, it is reasonable that an individual’s susceptibility towards the development of SD is ultimately dependent on their sensitivity to irritant unsaturated FFAs, e.g., oleic acid, and the subsequent inflammatory response [30,34]. This hypothesis is supported by the observation that oleic acid applied topically worsens skin shedding in patients with SD, while it has negligible consequences in unaffected subjects [7].

## 4. The Innate Immune System Involvement

Cutaneous innate immunity acts as the primary defense against the spread of pathogens from infection sites and is essential for activating the adaptive immune response, the body’s secondary defense mechanism.

During infections, pattern recognition receptors (PRRs) detect pathogen-associated molecular patterns (PAMPs) from invading viruses, thereby triggering antiviral innate immune responses. Notably, immunocompetent skin cells—including keratinocytes, Langerhans cells, dermal dendritic cells, macrophages, mast cells, and fibroblasts—possess PRRs to detect and respond to pathogens [35,36]. Different components of the innate immune system have been involved in SD pathogenesis and in *Malassezia* infection [37,38].

### 4.1. γδ T Cells and the MPZL3 Gene

γδ T cells are involved in immune-mediated skin diseases such as psoriasis and SD [39,40,41]. γδ T cells have unique properties and can be activated through cytokine signals [42]. In the context of SD, a study observed a significant increase in γδ T cells during the early-onset inflammation in a mouse model lacking Mpzl3 (myelin protein zero-like 3, a type I transmembrane protein). The absence of Mpzl3 in mice led to skin abnormalities and increased interleukin-17 (IL-17) levels primarily produced by γδ T cells [41]. Disruption of the epidermal barrier, coupled with the activation of the innate immune response and subsequent γδ T cell migration to the dermis, likely contributed to the SD-like symptoms.

The disruption of the epidermal barrier caused by the inactive Mpzl3 gene may facilitate the excessive proliferation of *Malassezia.* The overgrowth of *Malassezia* likely activates γδ T cells, which in turn promote the release of IL-17 [41]. Ruchti et al. demonstrated that *Malassezia* colonization of the skin is tightly regulated by the host immune system, particularly through Vγ4+ dermal γδ T cells, which rapidly expand and produce IL-17A to control fungal growth. Vγ4+ T cells can directly respond to *Malassezia*-derived ligands, which are shared across different *Malassezia* spp. [43].

Furthermore, a frameshift mutation in zinc finger 750 (ZNF750) has been associated with an SD-like phenotype in humans, with flaky lesions, and follicular hyperkeratotic plugs, all of which contribute to an overgrowth of *M. furfur* [44,45]. Hyperkeratosis and parakeratosis were apparent in skin biopsies. Mutations in the ZNF750 gene are associated with the impaired expression of late differentiation markers and skin barrier genes [46]. Understanding the ZNF750/MPZL3 pathway could provide insights into the pathogenesis of SD and identify new treatment targets.

### 4.2. NLRP3 Inflammasome Activation

Host germline-encoded PRRs initiate the initial immune response, recognizing conserved microbial motifs, endogenous damage signals, and pathogen-induced cellular alterations [47]. Among PRRs are accounted inflammasome-forming sensors that, upon activation, assemble into multimeric complexes [48].

**Figure 1:**
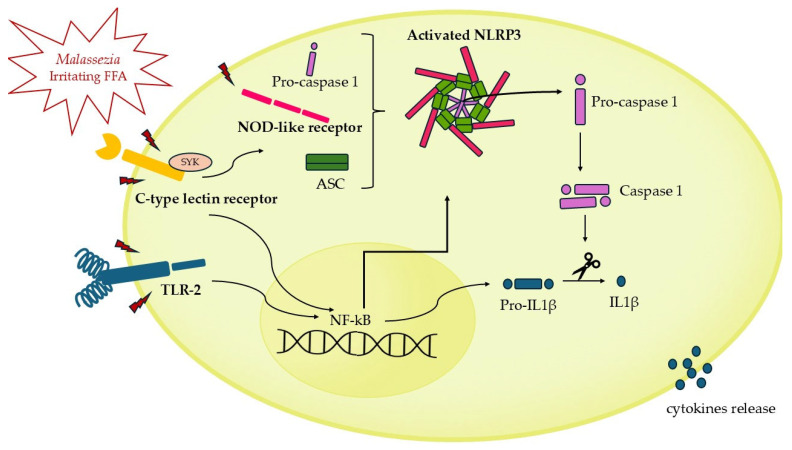
The assembly and activation of the NLRP3 inflammasome in response to NOD-like receptor (NLR) stimulated by *Malassezia* spp. and irritating FFAs. The inflammasome is assembled by three essential components: the sensor (NLR), the adaptor (ASC, apoptosis-associated speck-like protein containing a caspase recruitment domain), and the effector (caspase-1). Upon activation, caspase-1 interacts with clustered ASC leading to the formation of nucleated filaments. The activated caspase-1 cleaves pro-IL-1β to generate active IL-1β. TLR2 heterocomplex triggers NF-κB activation, which upregulates the expression of proinflammatory cytokines, including pro-IL-1β. Syk-coupled C-type lectin receptors activate both NF-κB and NLRP3 inflammasome. Upon NF-κB activation NLRP3 is upregulated, emphasizing the interplay between immune signaling pathways in the regulation of inflammation.

In susceptible individuals, *Malassezia* activates Antigen-Presenting Cells (APCs) via various PRRs, including Toll-like receptor-2 (TLR-2), NOD-like receptors, and C-type lectin receptors [49,50]. Notably, several clinical isolates of *Malassezia* spp., in particular *M. globosa* and *M. restricta*, [49,51] robustly activate the NLRP3 inflammasome, leading to the secretion of IL-1β (Figure 1). This is particularly significant in the pathogenesis of SD, as the activation of the NLRP3 inflammasome driven by *Malassezia* may play a central role in this condition. The resulting IL-1β secretion contributes to the chronic inflammation and hallmark symptoms of SD, such as erythema and scaling.

### 4.3. Mast Cells Potential Implication in Seborrheic Dermatitis

Mast cells (MCs) originate from pluripotent precursors in the bone marrow and circulate in the blood before maturing in various tissues, influenced by factors such as stem cell factor (SCF) and interleukin 3 (IL-3) [52,53,54]. MCs are prevalent in most body tissues, especially those serving as an interface between the host and environment, such as the skin and mucosal surfaces. Due to their distribution, MCs act as a frontline defense against external pathogens and environmental challenges [55,56].

MCs are central to many skin diseases due to their ability to release a wide range of mediators that influence inflammation and tissue remodeling. *M. sympodialis*, commonly found in atopic dermatitis, can activate MCs, leading to increased IgE-mediated degranulation through the TLR-2/MyD88 pathway [57]. Furthermore, MGL_1304, a protein from *M. globosa* detected in sweat, has been identified as an allergen capable of inducing IgE-dependent degranulation, highlighting the role of MCs in allergic responses in the skin [58].

The potential interaction between *Malassezia* spp., commonly associated with SD, allergic responses, and MCs suggests that these fungi may influence the disease process. Specifically, the crosslinking of the high-affinity IgE receptor (FcεRI) with antigen-bound IgE can trigger MC degranulation [57], which potentially exacerbates barrier dysfunction and amplifies the inflammatory responses.

Although MCs have been identified as a critical cellular component in the pathophysiology of certain dermatological conditions, there is currently no conclusive scientific evidence to substantiate their role in the immunological response observed in SD. However, given that MCs are the primary source of histamine in the human body [59] and that elevated histamine levels have been observed in SD patients [29,60], it is reasonable to assume that MCs play a role in the immunology of this disease. This hypothesis is further supported by the observation that histamine levels return to normal following the effective treatment of SD [29,49,61].

## 5. Adaptive Immunity

The skin-resident memory T cells, known as Trm cells, play a pivotal role in immune surveillance in boundary sites like the skin. They are non-circulating cells characterized by CD69, CD103, and CCR8 expression. Trm cells are involved in various skin pathologies and can influence Th1, Th2, and Th17 responses. Th1 cells are defined by their interleukin-2 (IL-2) and interferon-gamma (IFN-γ) production, while Th2 cells produce cytokines such as IL-4, IL-5, and IL-13. In SD, several studies have reported different findings regarding adaptive immune responses; some do not show any changes [62,63], while others indicate an increased immune response.

Molinero et al. demonstrated an upregulation of inflammatory and Th2 cytokines in skin biopsies from patients with SD, particularly in those with severe manifestations. Furthermore, the expression of the cytotoxicity-inducing MICA, a ligand for γδ-T lymphocytes, CD8+ T cells, and NK cells, was detected in the skin of SD patients using RT-PCR and hybridization with specific probes [64].

Another study conducted a comprehensive analysis of inflammatory cytokines, revealing elevated serum levels of IL-2 and IFN-γ in SD [65]. In addition, Corzo-León et al. highlighted the gene expression and proteomic responses of secreted cytokines in human skin exposed to *M. sympodialis*, characterized by high levels of TGF-β1 and IL-18 [66].

The role of IL-2 in regulating the immune response in SD may become particularly relevant. A case reported by Kawakami et al. illustrates an unusual adverse effect of systemic recombinant IL-2 (rIL-2) therapy: the development of SD-like eruptions and peri-orbital edema in a patient with renal cell carcinoma. A challenge test confirmed that these symptoms were induced by rIL-2, although the study did not explore the potential involvement of *Malassezia*. This suggests that IL-2 may contribute to SD-like symptoms through inflammatory pathways, though further investigation is needed to clarify this relationship [67].

## 6. Th17 and IL-17: Potential Link to Seborrheic Dermatitis?

Th17 cells and IL-17 play critical roles in autoimmune inflammation and maintaining tissue homeostasis. IL-17, mainly produced by various immune cells, regulates immune responses and is linked to inflammatory and autoimmune diseases. The mechanisms behind Th17 responses to *Malassezia* are not fully understood. It has been shown that C-type lectin receptors, such as Mincle, Dectin-1, and Dectin-2, recognize patterns in *Malassezia*’s cell wall and activate dendritic cells in vitro. However, in vivo studies have shown that Dectin-2 is the only receptor essential for initiating Th17 cell responses during skin colonization. Moreover, the Th17 response against *Malassezia* could depend on T-cell-intrinsic MyD88 signaling [68].

Recent studies have explored their involvement in atopic dermatitis, where they are supposed to play a vital role in immune activation and skin barrier function. Fang et al. found that higher levels of *Malassezia* in psoriasis correlate with increased LL37, IL-17A, TNF-α, and IL-23. The upregulation of LL37 by *Malassezia* promotes the IL-23/IL-17 pathway, which accelerates psoriasis severity [69]. Additionally, psoriasis lesions exhibit a more significant presence of Th17 cells than normal skin, with some Th17 cells producing TNF-α and IFN-γ. *Malassezia* activates the IL-23/IL-17 axis, which mediates the inflammatory response to the fungal infection [70]. Thus, targeting this pathway in therapies may inadvertently offer secondary protection against fungal infections. Andersen et al. found that certain HLA alleles may influence the risk of *Malassezia*-related skin diseases (MRSD). Additionally, they suggested that the genetic interaction between MHC class I and the IL-23/IL-17 pathway could play a role in MRSD, psoriasis, and atopic dermatitis. These findings suggest that *Malassezia* infections may require specific HLA presentation. However, further studies addressing microbial and protein changes are needed to clarify the immune mechanisms involved [71].

Considering the striking resemblance between psoriasis and SD [72,73], it is plausible that Th17 cells and IL-17 play a pivotal role in the etiology of SD [74]. The similarity of pathogenetic mechanisms indicates that Th17 signals may be involved in the inflammatory and immune responses underlying SD.

Nevertheless, further research is required to substantiate this hypothesis and to elucidate the precise mechanisms through which the IL-17/IL-23 axis may influence the pathogenesis and progression of SD. These studies may potentially lead to the development of novel targeted treatments, thereby improving the clinical management of patients with this condition.

## 7. Treatment and Future Perspectives

The management of SD includes the regulation of sebum secretion, limiting colonization of the fungus *Malassezia* spp., and mitigating inflammation. Sebum production can be controlled with different strategies, such as medicated shampoos or topical treatments. Limiting the colonization of *Malassezia* spp. may be achieved with antifungal agents. Corticosteroids or other anti-inflammatory agents are frequently prescribed for people with SD. Less severe cases of SD are treated topically, in most cases. Severe cases are often managed with off-label topical and systemic medications (Table 1) [1]. Recent studies have highlighted the alteration of the microbiome in people with SD. One study demonstrated that the administration of recombinant human thymosin beta-4 (rhTβ4) led to a significant improvement in scalp condition and microbiome homeostasis [75]. Additionally, the modulation of the skin mycobiome with the administration of a probiotic-enriched oily suspension has been proposed as a treatment for SD [76].

### 7.1. Topical Antifungal Agents

Antifungal treatments have been proven to be effective in managing SD by reducing yeast populations and controlling inflammation [77]. Topical antifungals, such as ketoconazole, clotrimazole, and miconazole, are often first-line treatments for SD because they inhibit fungal cell wall synthesis [78]. Ketoconazole, introduced in 1979, acts by blocking ergosterol synthesis and has additional anti-inflammatory effects [79,80]. Miconazole is notable for its efficacy and safety comparable to ketoconazole [81], while clotrimazole 1% cream is a broad-spectrum option that is generally well tolerated but occasionally causes localized irritation [82]. Ciclopirox olamine 1% is another broad-spectrum antifungal that inhibits metal-dependent enzymes in fungi and offers anti-inflammatory benefits [78,83]. Despite their efficacy, these treatments have been shown to induce resistance in *Malassezia* strains. This suggests that topical therapies alone may not always be sufficient [29,84].

### 7.2. Systemic Antifungal Agents

Systemic antifungal treatments, such as terbinafine and itraconazole, are typically reserved for severe, acute, or treatment-resistant cases of SD, and for conditions that are particularly challenging to manage [1]. Systemic therapy primarily aims to alleviate symptoms rapidly before the subsequent use of topical treatments in the long-term maintenance of the condition. Terbinafine is an antifungal medication with additional antioxidant and anti-inflammatory properties. Oral terbinafine has been demonstrated to be effective in managing SD [82,85]. Itraconazole is a triazole antifungal that is highly effective in the keratin- and lipid-rich environment of the stratum corneum, where *Malassezia* typically resides [86]. In addition to its antifungal action, itraconazole has anti-inflammatory effects due to inhibition of the formation of 5-lipoxygenase metabolites, which are inflammatory mediators [1,87]

### 7.3. Anti-Inflammatory Agents

Topical corticosteroids, ranging from potent (e.g., betamethasone valerate) to super potent (e.g., clobetasol propionate), are crucial in the effective management of SD symptoms such as erythema, scaling, and itching through their anti-inflammatory, immunosuppressive, and antiproliferative activities [1,88]. Non-steroidal anti-inflammatory agents offer significant benefits in managing both scalp and non-scalp SD by inhibiting *Malassezia* through their anti-inflammatory, antimycotic, keratolytic, and antioxidant properties [78,89]. These treatments are generally well-tolerated, with only mild side effects [78,90,91]. Immunomodulators, specifically tacrolimus [83,92,93] and pimecrolimus [78,94,95,96,97], are effective in the treatment of facial SD due to their limited side effects. These drugs inhibit calcineurin, essential for T cell activation and the production of pro-inflammatory cytokines, and may also exhibit antifungal activity against *Malassezia*. These agents are beneficial when topical corticosteroids and antifungals do not control SD adequately. When conventional treatments are insufficient, as in cases of severe SD, managing CD4+ levels with antiretroviral medications can be advantageous. The prevalence of SD in patients with neurological conditions, such as Parkinson’s disease, often presents as unilateral facial dermatitis and indicates a need for targeted treatments, including antifungals and anti-inflammatory agents [1].

## 8. Conclusions

In this review, we delved into the intricate role of the immune system in the development of SD, particularly focusing on the interaction between *Malassezia* and various components of the immune response. Through an in-depth analysis of the existing literature, we have highlighted how skin barrier impairment, innate immunity, and adaptive immunity contribute to the clinical manifestation of SD.

We emphasized how alterations in the skin barrier can facilitate fungal colonization and trigger immune responses, contributing to the disease’s pathogenesis. The importance of the innate immune response, particularly through γδ T cells, MCs, and the NLRP3 inflammasome, emerged as a central factor in amplifying skin inflammation.

Additionally, we explored the role of adaptive immunity, focusing on Th1, Th2, and Th17 cells, suggesting that excessive immune reactivity and an altered immune balance may play a pivotal role in the pathogenesis of SD. Clinical and experimental studies have demonstrated that the immune response is markedly altered in patients with SD. The presence of a hyper-reactive innate immune response, excessive production of pro-inflammatory cytokines, and alterations in Th1 and Th2 responses provide evidence to support an immune-mediated pathogenesis. The current literature also suggests that Th17 cells, known to be implicated in other skin conditions, may also play a role in the pathogenesis of SD.

**Figure 2:**
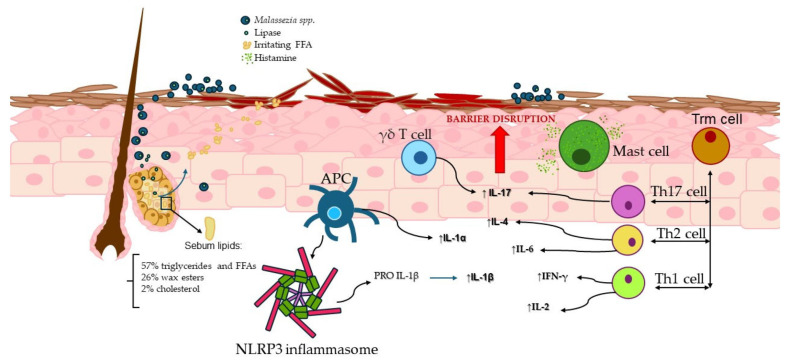
A hypothetical pathogenic pathway involving the colonization of *Malassezia* spp. under immunocompromised conditions. The lipase produced by the fungus acts on sebaceous lipids, resulting in the release of irritating FFAs. Dendritic cells, γδT cells, MCs, and Trm cells thus initiate an innate immune response. Activated dendritic cells trigger the NLRP3 inflammasome, which contributes to the production of IL-1β. MCs release histamine, inducing pruritus. Trm cells also involved in memory recall, activate Th1, Th2, and Th17 subsets, which produce IL-4, IL-6, IFN-γ, IL-2, and IL-17.

A hypothetical pathogenic pathway may begin with the colonization of *Malassezia* spp. under immunocompromised conditions. The lipase produced by the fungus acts on sebaceous lipids, resulting in the release of irritating FFAs. Dendritic cells, γδT cells, MCs, and Trm T cells are activated by *Malassezia*, initiating an innate immune response. Activated dendritic cells trigger NLRP3, which contributes to the production of IL-1β. MCs release histamine, inducing pruritus. Trm cells, involved in the initial immune response and memory recall, activate Th1, Th2, and Th17 subsets, which produce IL-4, IL-6, IFN-γ, IL-2, and IL-17. These processes ultimately result in the deterioration of the skin barrier, which in turn exacerbates and perpetuates the underlying symptoms. This entire mechanism is described in Figure 2.

In conclusion the review underscores how a deeper understanding of the relationship between *Malassezia* and the immune system, as well as the central role of inflammation, could guide the development of targeted therapeutic strategies, particularly when various treatment approaches, including both antifungal agents and immunomodulators, are available, thereby enhancing the management of SD and the quality of life of patients.

## Figures and Tables

**Table 1:** Overview of treatments for SD. The table summarizes various therapeutic approaches such as antifungals, corticosteroids, topical treatments, and immunomodulators, with their specific mechanisms of action and their respective indications and recommended use.

Treatment Category	Therapy	Mechanism of Action	Medical Indications
Topical anti-fungal agents	Ketoconazole Miconazole Clottrimazole 1% Ciclopiroxolamine 1%	Inhibition of Ianosterol 14-a demethylase Depletion of ergosterol Toxic sterols in membrane	Mild to moderate SD
Systemic anti-fungal agents	Terbinafine Itraconazole	Inhibition of ergosterol synthesis Inhibition of squalene epoxidase	Mild to moderate SD
		Inhibition of 5-lipoxygenase metabolites	Moderate to severe SD
Anti-inflammatory agents	Non-steroidal (ciclopirox7piroctonolamine)	Inhibition of P450-mediated reactions	Mild to moderate SD
	Topical corticosteroids (betamethasone valerate, clobetasol propionate)	Anti-inflammatory Immunosuppressive Antiproliferative	Moderate to severe SD
Immunomodulators	Tacrolimus Picrolimus	Inhibition of calcineurin	Mild to moderate SD (short-term treatment)

## Abbreviations

The following abbreviations are used in this manuscript:

APC	Antigen-presenting cell
FFA	Free fatty acid
FAS	Fatty acid synthase
HIV/AIDS	Human Immunodeficiency Virus/Acquired Immunodeficiency Syndrome
IFNg	Interferon-gamma
IL-17	Interleukin 17
MC	Mast cell
MRSD	Malassezia-related skin disease
PAMPs	Pathogen-associated molecular patterns
PRRs	Pattern recognition receptors
SCF	Stem cell factor
SD	Seborrheic dermatitis
SGs	Sebaceous glands
TLR2	Toll-like receptor 2
TEWL	Transepidermal water loss
ZNF750	Zinc finger 750
